# Longitudinal extensive transverse myelitis with an abnormal uFLC ratio in a pediatric patient: Case report and literature review

**DOI:** 10.1097/MD.0000000000009389

**Published:** 2017-12-29

**Authors:** Po-Chang Hsu, Shyi-Jou Chen

**Affiliations:** a Department of Pediatrics, Tri-Service General Hospital; b Department of Microbiology and Immunology, National Defense Medical Center, Taipei, Taiwan.

**Keywords:** acute transverse myelitis, free light chains, longitudinally extensive transverse myelitis, monoclonal gammopathy of undetermined significance, pediatric

## Abstract

**Rationale:**

The serum and urine-free light chain (sFLC/uFLC) ratios of kappa (κ) to lambda (λ) serve as biomarkers for plasma cell disorders, especially multiple myeloma. However, to our best knowledge, the ratios have not been appropriately assessed for acute transverse myelitis (ATM).

**Patient concerns:**

We present a 12-year-old boy who had sudden onset low back pain following paralysis of his 4 extremities and disturbance consciousness. Magnetic resonance imaging (MRI) of the brain and spine indicated diffuse hyperintensity in T2-weighted images from the cervical spinal cord to the conus medullaris. An abnormal serum M-peak and uFLC ratio were detected in acute stage.

**Diagnoses:**

Based on the image findings, laboratory findings, and physical examination results, the diagnosis of acute transverse myelitis was established.

**Interventions and outcomes:**

With the treatment of pulse therapy and 5 courses of plasmapheresis, the patient had improvement in expanded disability status scale (EDSS) score from 9 to 5. Besides, the κ/λ ratio was also returned within the normal range.

**Lesson:**

The case presented an unusual phenomenon of transient abnormal κ/λ ratio combined with an M-peak in the acute course of longitudinally extensive transverse myelitis (LETM), which revealed FLC ratio recovering accompany with the improvement of disease. Further studies are required to identify the association between ATM and monoclonal gammopathy of undetermined significance (MGUS).

## 1 Introduction

Acute transverse myelitis (ATM) is an immune-mediated neurological disorder of the spinal cord,^[1]^ and longitudinally Extensive Transverse Myelitis (LETM) is defined as an inflammation affecting the spinal cord and extending over 3 or more vertebral segments.^[2]^ Rarely, it may present with factors that may confound the diagnosis. Free light chains (FLCs) are important disease biomarkers in patients with plasma cell-proliferative disorders, which produce large amounts of abnormal monoclonal immunoglobulins. The concentrations of kappa (κ) and lambda (λ) light chains can be elevated during inflammation or renal impairment, but the κ/λ ratio remains unchanged. In contrast, an M-peak with an abnormal κ/λ ratio usually indicates plasma cell disorders. For urine FLC (uFLC) and serum FLC (sFLC) testing, both κ and λ are measured to calculate the κ/λ ratio, which can help detect, diagnose, and monitor plasma cell disorders, including multiple myeloma (MM), primary amyloidosis, and monoclonal gammopathy of undetermined significance (MGUS).^[3]^ Here, we report a 12-year-old boy with ATM associated with an M-peak and an elevated urine κ/λ ratio. To our knowledge, such a condition has not been reported previously.

## 2 Ethic statement

The study was approved by the Institutional Review Board of Tri-Service General Hospital (TSGH-IRB, approval number: 2-106-05-091). Informed consent was obtained from the patient's parents for the publication of this case report.

## 3 Case presentation

The patient was a 12-year-old boy. He had no medical history or specific family history. He did not receive vaccination within the 3 months prior to disease onset and did not experience any preceding infection or trauma. He presented with sudden onset low back pain and left upper limb weakness following paralysis and numbness of his 4 extremities and disturbance consciousness. He was intubated and placed on mechanical ventilation for airway protection. Complete blood count and comprehensive metabolic panels were normal. Computed tomography of his head without intravenous contrast showed no hemorrhage or midline shift. His consciousness recovered gradually with clear mental status, but hypotonia was sustained in all 4 limbs, and hyperalgesia and flaccid bladder associated with constipation were still noted.

On physical examination, his consciousness was clear, and cranial nerve examination, including eye fundoscopy, was normal. Pupils were equal in size, round, and reactive. No afferent pupillary defect was noted. Extraocular movements were full. There was no nystagmus and no internuclear ophthalmoplegia. Face sensation was normal. Face was symmetric. Hearing was intact. Tongue and uvula were midline. Sensory examination was notable for normal proprioception and vibration throughout. There was decreased temperature, pinprick, and hyperesthesia below his neck, with a C4 sensory level noted. His strength was 0/5 in all 4 extremities, and deep tendon reflexes were increased with ankle clonus and sensory disturbance. Hyperesthesia below the neck was found. Sensation and movement were preserved over the head and neck.

**Figure 1:**
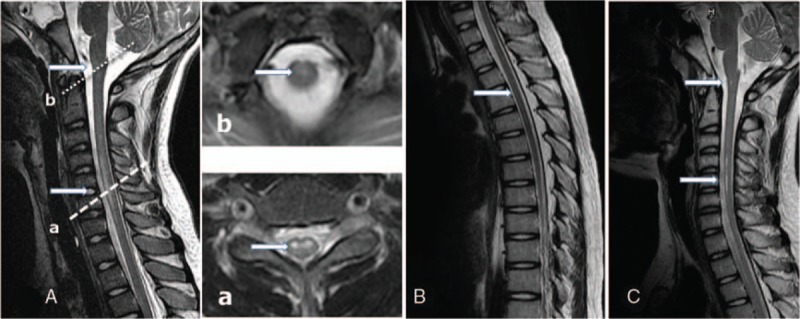
Serial MR images of T2-weighted images. A, At admission, longitudinally extensive cord lesions mainly involving the gray matter with hyperintensity and swelling from thoracic to cervical spinal cord (T1-C3) and conus medullaris (a, b). B, Two months later after the onset, diffused increased signal intensity on T2WI from cervical spinal cord to conus medullaris without enlargement or atrophy. C, Five months later after the onset, small dot and linear-like cavities are seen in the cervical spine and cervicomedullary junction. MR = magnetic resonance.

Cerebrospinal fluid (CSF) analysis showed normal values for white blood cell count and protein and glucose levels, and negative results for bacterial culture and virus polymerase chain reaction (PCR). Magnetic resonance imaging (MRI) of the brain and spine (Fig. 1A) indicated diffuse hyperintensity in T2-weighted images from the cervical spinal cord to the conus medullaris, consistent with transverse myelitis. Tests for other associated conditions, such as lupus, botulism, antiphospholipid antibodies, influenza, mycoplasma antibodies, and HIV (quantitative RT-PCR), were negative. The patient was started on methylprednisone pulse therapy, but he showed only a slight response at another local hospital. Two weeks after disease onset, he was referred to our hospital for further evaluation.

**Figure 2:**
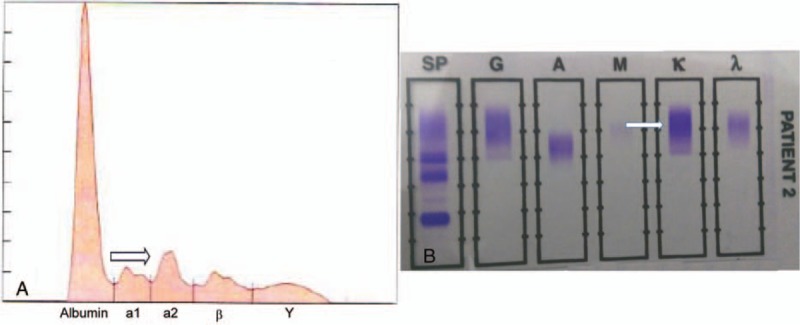
Serum electrophoresis. A, A monoclonal peak is observed in the Alpha 2 fraction in serum protein electrophoresis. B, Kappa (κ), lanes were identified in serum immunofixation electrophoresis. λ = lambda, A = IgA, G = IgG, M = IgM, SP = standard protein.

Lumbar puncture was repeated at our hospital. The cell count was 6/uL, protein level was 29 mg/dL, and glucose level was 56 mg/dL. CSF aerobic and fungal cultures, enterovirus RT-PCR, venereal disease research laboratory, Aquaporin-4 IgG, throat swab, and stool assessment for enterovirus (PCR) were negative. Serum and CSF oligoclonal bands were also absent, but immunoglobulin electrophoresis (IE) (Fig. 2) revealed increased α2-globulins (<3 g/dL) with an M-peak presentation. Hence, 24-hours urine was collected, and the κ/λ ratio was found to be 19.23, the excretion of urinary protein was 156 mg/24 h. However, the patient presented without hypercalcemia, renal dysfunction, anemia, or lytic bone lesions.

**Figure 3:**
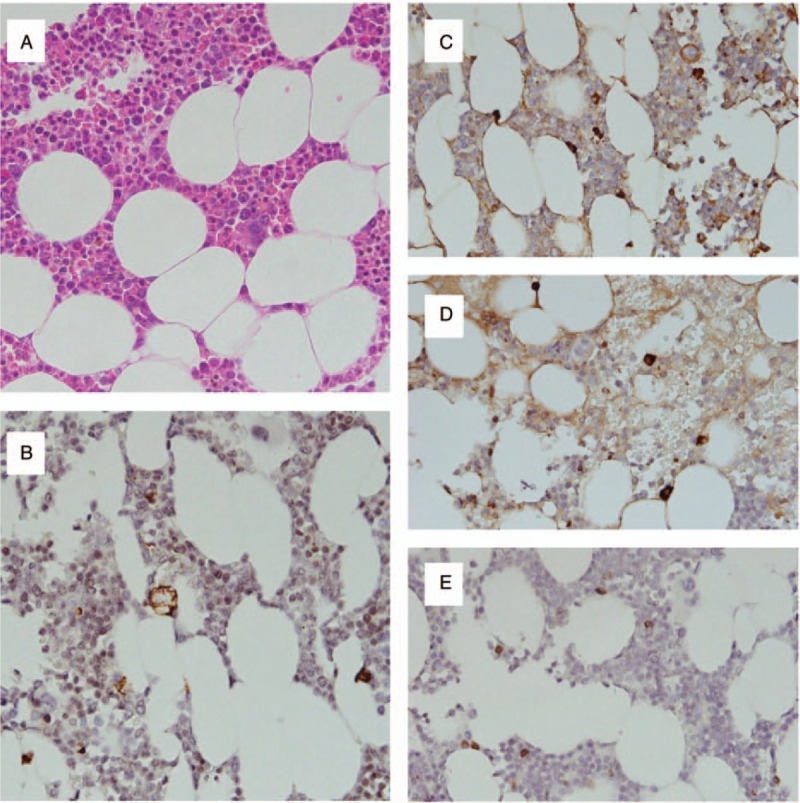
Patient's bone marrow biopsy. A, Hypocellular bone marrow aspirate smear (×400). B, Immunostaining for CD138 showed 2% CD138 positive cells. C and D, Immunostaining for free kappa light chains (C) and free lambda light chains (D) showed no kappa nor lambda light chain restriction pattern. E, Immunostaining for CD79a showed negative for CD79a.

After repeat lumbar puncture, the patient underwent plasmapheresis (PLEX) every other day for 5 sessions, with improvement in the expanded disability status scale (EDSS) score from 9 to 7. Two weeks after the completion of PLEX, repeat IE and bone marrow (BM) aspiration were performed. The free κ/λ ratios of serum and urine were 0.34 and 0.32, respectively. The BM (Fig. 3) revealed only hypocellularity and no increased plasma cell count (2%). The patient was discharged after BM aspiration, and he was readmitted for repeat MRI 2 months (Fig. 1B) and 5 months (Fig. 1C) later. Both MRI scans showed partial improvement of myelopathy with minimal persistent signal enhancement in T2-weighted images and diffusion-weighted images.

Of note, during his hospital stay, the function of the 4 limbs improved after PLEX, and his bladder and rectal disturbances resolved. At about 6 months after PLEX, he was able to eat with chopsticks by himself and ambulate with support, and his EDSS score was 5.

## 4 Discussion

Pediatric ATM is an immune-mediated CNS disorder classically described as demyelinating, and the major tool for diagnosis and prognosis is MRI. Since the involved spinal cord is more than 3 vertebral segments in our patient, it is not only ATM but also LETM. Additionally, ATM is an exclusion diagnosis, which may be the first presentation of relapsing acquired demyelinating syndromes or an autoimmune rheumatologic disorder.^[1]^ The abnormal M-peak and FLC ratio in our case were surprising because they are not a phenomena known to be associated with ATM.

FLCs are important disease biomarkers in patients with plasma cell-proliferative disorders.^[4]^ uFLC testing is performed to help detect, diagnose, and monitor plasma cell disorders, including MM, primary amyloidosis, and MGUS, and to monitor the effectiveness of treatment. The etiology of an abnormal FLC ratio, including MM and related plasma disorders, is well described, but the association between FLCs and ATM has not been reported previously.

MGUS has been reported in association with several nonmalignant disorders, and it has been reported along with autoimmune disorders (Table 1).^[5–15]^ However, the exact mechanism is not clear, and it is not clear whether these conditions are pathogenetically related or merely represent coincidental associations. There were some differences between our case and the previous cases. First, our case presented with transient MGUS, while other cases presented with MGUS for more than 2 years. Second, our case did not show clinical manifestations associated with plasma cell myeloma. Third, this is the first case of ATM associated with MGUS. Finally, our patient was a 12-year-old boy, while previous patients with MGUS were usually above the age of 50 years.

**Table 1:**
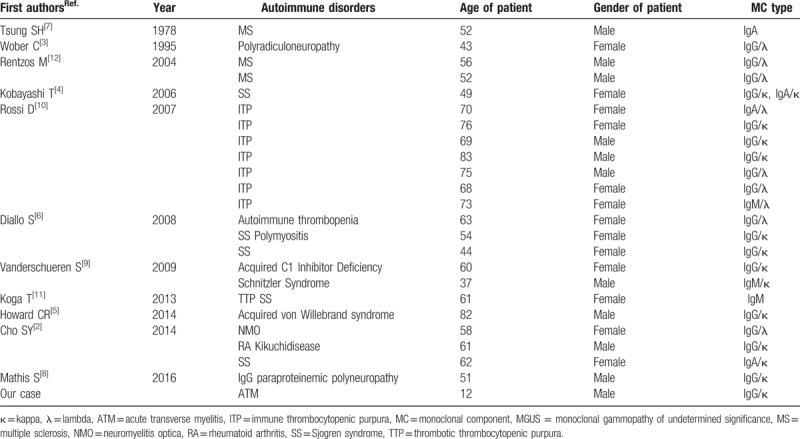
Reported cases of MGUS associated autoimmune disorders.

A series of studies^[1,16]^ on ATM have been reported to define the risk factors for outcomes, but the prognosis still presents a challenge because of the inconsistent use of core outcomes. A retrospective study on ATM in childhood involving 47 patients revealed the factors associated with a worse functional outcome, including younger age, longer time from symptom onset to treatment, longer segmental involvement, higher spinal level, presence of T1-hypointense lesions, and a high number of white blood cells in the CSF.^[16]^ CSF analysis and serum studies have shown biomarkers, including aquaporin-4 antibody, intrathecal oligoclonal bands, and virus presence (PCR), to distinguish specific etiologies from isolated ATM, but there is no independent predictor for ATM.^[1]^

A correlation between CSF interleukin-6 levels and disability in patients with ATM has been reported, but an abnormal FLC ratio has not been documented till date.^[17]^ FLCs have been reported as powerful prognostic markers for non-neoplastic disorders,^[18–20]^ and have been considered significant predictors of worse overall survival in the general population of persons without plasma cell disorders.^[21]^ Bellary et al^[18]^ reported that sFLCs increased the risk of CVD events in type 2 diabetes. Deng et al^[19]^ showed that the elevation of sFLCs not only preceded the development of the disorder but also was associated with mortality among patients with RA. Hutchison et al^[20]^ described FLCs as factors that independently predict mortality in people with CKD.

In this case, increased α2-globulins with an M-peak and elevated uFLC ratio were found initially and declined as the severity of the disease subsided. To our knowledge, this is the first case of ATM with an M-peak and abnormal uFLC ratio. The recovered IE and ratio between the κ and λ light chains, along with improved clinic symptoms, support the proposition that the FLC ratio can be used as an indication of disease progression or remission.

We considered the possibility that the FLC ratio may be falsely elevated owing to other etiologies, but the result of BM examination and the serum immunoglobulin level ruled out the possibility of a coexisting plasma cell disorder. Urine and serum concentrations of FLCs are dependent upon the balance between production and renal clearance. sFLCs are rapidly cleared through the renal glomeruli with half-lives of between 2 and 6 hours before being metabolized in the proximal tubules of the nephrons. Hence, the uFLC ratio in our case was high before PLEX and declined at 2 weeks after the treatment, which would not be expected if the decline in FLCs was due to PLEX.

We did not assess the sFLCs before PLEX, and an inflammatory condition may influence the results. However, a previous study demonstrated that serum κ and λ concentrations of 133 and 278 mg/L, respectively, are required to allow the detection of FLCs in urine,^[22]^ and a recent study demonstrated that pediatric patients with inflammatory conditions showed no change in the uFLC ratio.^[23]^ Therefore, we believe that our experience highlights the uncommon features at presentation in a relatively common immune-mediated CNS disorder. The association described herein between the FLC ratio and ATM identifies the FLC ratio as a biomarker of potential clinical utility. Moreover, FLC assessment is more accessible than CSF assessment and more acceptable by a patient's family owing to the involvement of a less invasive procedure.

## 5 Conclusion

This case report presents an unreported phenomenon of LTEM with an abnormal uFLC ratio in a pediatric patient. To our knowledge, presentation with MGUS in ATM in such a young patient has not been reported previously. We will follow the patient's condition to assess the risk of progression to related malignant neoplasm or other coincidental diseases. Further studies are required to identify the association between ATM and MGUS, as well as to determine the mechanistic basis for this association.

To conclude, this case highlights the need of meticulous observation and exploration of the FLC ratio in the cases of ATM for the better understanding of the phenomena and the association with the dependence syndrome.

## References

[1] Absoud M Greenberg BM Lim M Pediatric transverse myelitis. Neurology 2016;87 (9 suppl 2):S46–52. 27572861 10.1212/WNL.0000000000002820

[2] Wingerchuk DM Hogancamp WF O’Brien PC The clinical course of neuromyelitis optica (Devic's syndrome). Neurology 1999;53:1107–14. 10496275 10.1212/wnl.53.5.1107

[3] Tosi P Tomassetti S Merli A Serum free light-chain assay for the detection and monitoring of multiple myeloma and related conditions. Ther Adv Hematol 2013;4:37–41. 23610612 10.1177/2040620712466863 PMC3629763

[4] Jenner E Serum free light chains in clinical laboratory diagnostics. Clin Chim Acta 2014;427:15–20. 23999048 10.1016/j.cca.2013.08.018

[5] Cho SY Yang HS Jeon YL A case series of autoimmune diseases accompanied by incidentally diagnosed monoclonal gammopathy: is there a link between the two diseases? Int J Rheum Dis 2014;17:635–9. 24460798 10.1111/1756-185X.12267

[6] Wober C Schmidbauer M Podreka I Chronic relapsing polyradiculoneuropathy in IgG lambda monoclonal gammopathy of undetermined significance (MGUS)—complete remission following carmustine treatment. Wien Klin Wochenschr 1995;107:318–20. 7785279

[7] Kobayashi T Muto S Nemoto J Fanconi's syndrome and distal (type 1) renal tubular acidosis in a patient with primary Sjogren's syndrome with monoclonal gammopathy of undetermined significance. Clin Nephrol 2006;65:427–32. 16792139 10.5414/cnp65427

[8] Howard CR Lin TL Cunningham MT IgG kappa monoclonal gammopathy of undetermined significance presenting as acquired type III Von Willebrand syndrome. Blood Coagul Fibrinolysis 2014;25:631–3. 24686099 10.1097/MBC.0000000000000112 PMC4119490

[9] Diallo S Ndiaye FS Pouye A [Monoclonal gammapathy of undetermined significance and autoimmune disease: description of three cases in Senegal]. Med Trop (Mars) 2008;68:65–8. 18478776

[10] Tsung SH Monoclonal gammopathy associated with multiple sclerosis. Ann Clin Lab Sci 1978;8:472–5. 736511

[11] Mathis S Franques J Richard L Monoclonal gammopathy of undeterminated significance and endoneurial IgG deposition: a case report. Medicine 2016;95:e4807. 27603395 10.1097/MD.0000000000004807 PMC5023918

[12] Vanderschueren S Mylle M Dierickx D Monoclonal gammopathy of undetermined significance: significant beyond hematology. Mayo Clin Proc 2009;84:842–5. 19720783 10.4065/84.9.842 PMC2735435

[13] Rossi D De Paoli L Franceschetti S Prevalence and clinical characteristics of immune thrombocytopenic purpura in a cohort of monoclonal gammopathy of uncertain significance. Br J Haematol 2007;138:249–52. 17535272 10.1111/j.1365-2141.2007.06633.x

[14] Koga T Yamasaki S Nakamura H Renal thrombotic microangiopathies/thrombotic thrombocytopenic purpura in a patient with primary Sjogren's syndrome complicated with IgM monoclonal gammopathy of undetermined significance. Rheumatol Int 2013;33:227–30. 20652270 10.1007/s00296-010-1569-0

[15] Rentzos M Michalopoulou M Gotosidis K Unusual association of multiple sclerosis with monoclonal gammopathy of undetermined significance (MGUS): two case reports. Funct Neurol 2004;19:253–6. 15776794

[16] Pidcock FS Krishnan C Crawford TO Acute transverse myelitis in childhood: center-based analysis of 47 cases. Neurology 2007;68:1474–80. 17470749 10.1212/01.wnl.0000260609.11357.6f

[17] Kaplin AI Deshpande DM Scott E IL-6 induces regionally selective spinal cord injury in patients with the neuroinflammatory disorder transverse myelitis. J Clin Invest 2005;115:2731–41. 16184194 10.1172/JCI25141 PMC1224298

[18] Bellary S Faint JM Assi LK Elevated serum free light chains predict cardiovascular events in type 2 diabetes. Diabetes Care 2014;37:2028–30. 24742658 10.2337/dc13-2227

[19] Deng X Crowson CS Rajkumar SV Elevation of serum immunoglobulin free light chains during the preclinical period of rheumatoid arthritis. J Rheumatol 2015;42:181–7. 25593227 10.3899/jrheum.140543 PMC4371213

[20] Hutchison CA Burmeister A Harding SJ Serum polyclonal immunoglobulin free light chain levels predict mortality in people with chronic kidney disease. Mayo Clin Proc 2014;89:615–22. 24797643 10.1016/j.mayocp.2014.01.028

[21] Dispenzieri A Katzmann JA Kyle RA Use of nonclonal serum immunoglobulin free light chains to predict overall survival in the general population. Mayo ClinProc 2012;87:517–23. 10.1016/j.mayocp.2012.03.009 PMC3538473 22677072

[22] Nowrousian MR Brandhorst D Sammet C Serum free light chain analysis and urine immunofixation electrophoresis in patients with multiple myeloma. Clin Cancer Res 2005;11 (24 pt 1):8706–14. 16361557 10.1158/1078-0432.CCR-05-0486

[23] Cho SY Nam YS Yang JJ Free light chain levels in pediatric patients with inflammatory conditions. Clin Lab 2014;60:1245–8. 25134398 10.7754/clin.lab.2013.130819

